# The Doctor in History

**Published:** 1890-03-29

**Authors:** 


### The Doctor in History.

Among those who have had the making of history, doctors have played a far more important part than has usually been attributed to them. They are some of them not without their influence now. It seems but the other day, indeed, that peace or war in Europe appeared to be depending on the result of a surgical operation, and every day medical men must be having more or less completely in their keeping, lives, the losing or saving of which must exert the most momentous effect on the course of public events.

They are still potent magicians in some situations, but they do not take that heavy and ubiquitous share in politics that they did when Charles II. recognised in the society physicians of his day one of the most troublesome of political forces. Nell Gwynn's doctor, said this good-humoured cynic, did him more mischief than a troop of horse could do. This meddlesome practitioner was Doctor Lower, a particularly stiff-backed Protestant, who, at the time when the air was full of rumours of Catholic intrigue, in which the King himself was the prime instigator, would so cleverly draw the vivacious and unsuspecting Nelly as to obtain a tolerably complete knowledge of all that was going on behind the scenes at Court. "He would fish out of her," as our chronicler records, "all the intrigues of the Court of King Charles." The doctors, indeed, keep cropping up in all the records of the gossips of the times, and it is easy to see what a very influential part they must have played in those days of intrigue and plotting and scheming. "Oct. 24th, 1662, Mr. Pierce, the chyrurgean," says Samuel Pepys, "tells me how all things go at Court; that the king do show no countenance to any that belong to the Queen . . . That her owne physician did tell him within these three days that the Queen do know how the King order things, and how he carries himself to my Lady Castlemaine and others, as well as anybody; but though she hath spirit enough, yet seeing that she do no good by taking notice of it, for the present she ponders it in policy."

In the seventeenth century, indeed, doctors seem to have played pretty much the part of the "society journalism" of our own day, though not infrequently they took more than a gossipping share in the Court and political complications of their times. Pepys shows us that it was an army physician who carried on negotiations between General Monk and Charles II. "This evening came Dr. Clarges to Deale, going to the King; when the Townes-people strewed the streets with herbes against his coming for joy of his going. Never was there so general a content as there is now." There were, it seems, three "doctors of physique" in the King's train when he came to England, and it is, by the way, very amusing to observe the versatile and inquistive Pepys chatting with one of them, even on so exciting an occasion, on some of the newly-originated ideas about human eye-sight. "I put Dr. Scarborough in mind of what I heard him say, that children do in every day's experience look several ways with both their eyes, till custom teaches them otherwise. And that we do now see but with one eye, our eyes looking in parallel lines."

Talking of these three physicians coming over in the King's train of courtiers reminds one of the historically-recorded fact that with the army that went over with Henry V. to invade the kingdom of France, and which fought the battle of Agincourt, there was but a solitary surgeon in attendance. If we may infer from this fact anything about the usual surgical arrangements of mediæval armies, what a frightful idea it affords of those battle-fields, many of which were really more sanguinary than the average modern ones. Only a single surgeon to an invading army, and that surgeon equipped only with fifteenth-century appliances and fifteenth-century science!

If the somewhat gushing testimony of James II. at the time of the birth of a son could be taken quite seriously, few men have exerted a more momentous influence in modern history than Dr. Walgrove, when, as the king seems to have believed, he saved the life of Mary Beatrice of Modena and of her newly-born son. The joyful King commanded the physician to kneel down by the Queen's bedside, and then and there dubbed him a knight. The delight of the King was unbounded, but perhaps he was a little precipitate in bestowing honours, for within a few hours the chief physician and some of his colleagues between them managed to come within an ace of killing the infant prince. "The physicians," afterwards wrote the royal mother, "prescribed something for him which they say is good for babies. I don't know how it was; but this I know, that by mistake or carelessness they repeated the dose, which made him so ill that everyone thought he was dying." There were some who believed that he actually did die, and that another infant was substituted by Sir William Walgrove, who, indeed, has been credited by some with having done his best to control the current of events by smuggling in a child in the first instance by means of a warming-pan. There can be little doubt, however, that all such stories were groundless. James seems to have thought his throne was now secure, and that his troubles were all over. Alas! for him. The birth of that ill-starred son just precipitated the revolution which would, perhaps, have broken out long before only that the people of England had apparently made up their minds that, as the King had no son, and the crown would, on his decease, go to the Protestant Mary of Orange, it would be better to endure as patiently as they could what remained of the King's rule.

For some time, however, it seemed to be very doubtful whether the little stranger would live to take any share in personal rivalries. Death and the doctors seemed to be fighting a desperate battle over the royal cot, and the revolutionary outburst was delayed till the contest was decided. The king himself made light of little night alarm when it was over. "The child was somewhat ill last night of the wind," wrote King James to his son-in-law, "but is now, bleased be God, very well again, and like to have no return of it, and is a very strong boy." It is curious to learn what was the kind of fare that the united wisdom of six of the best doctors of the day could devise for a young prince, on whom the future of a dynasty depended, two centuries ago. "This morning", writes the Papal Nuncio, at the Court of St. James's, "I have had the honour of seeing him whilst they gave him his food which he took with a good appetite. He appears to me very well complexioned and well made. The said aliment is called Watter Gruell; it is compounded of barley flour, water and sugar to which a few currants are sometimes added."

On this right royal fare the unhappy little creature soon began to suffer greatly from indigestion and inflammation of the bowels, and in about six weeks they thought every sigh would be his last. The six learned doctors shook their wise heads and gave him up for dead, and the Queen, in her maternal agony, desperately took matters into her own hands. She sent for a wet nurse, to whom the child took with avidity, and began to mend with the first meal. The child thus saved by the wife of a poor tile-maker, of Richmond, lived to be the "Old Pretender."

Before the little one was more than about six months old we find his mother climbing on board a vessel at Gravesend in the guise of an Italian washerwoman with the Prince of Wales tucked under her arm in a bundle that looked like linen. Both the father and mother of the unlucky young prince afterwards expressed their conviction that it was only by something very like a miracle that the child did not cry and so prevent the escape. But Sir William Walgrove—the physician who had been knighted by the Queen's bedside—was in the train of the fugitive, and probably the worthy doctor had more to do with the convenient slumbers of the Prince of Wales at that momentous juncture than history has recorded.

